# Cross-cultural adaptation and validation of the Arabic version of the functional index for hand osteoarthritis

**DOI:** 10.1186/s12891-020-03418-8

**Published:** 2020-06-19

**Authors:** Fatima Zahrae Taik, Latifa Tahiri, Hanan Rkain, Ilham Aachari, Emmanuel Maheu, Fadoua Allali

**Affiliations:** 1 Department of Rheumatology B, El Ayachi Hospital, Ibn Sina University Hospital, Rabat, Morocco; 2 Faculty of Medicine and Pharmacy, Mohammed V University, Rabat, Morocco; 3 Laboratory of Physiology, Faculty of Medicine and Pharmacy, Mohammed V University, Rabat, Morocco; 4 Department of Rheumatology, Saint-Antoine Hospital, Paris, France

**Keywords:** Osteoarthritis, Hand, FIHOA, Validation, Psychometric properties

## Abstract

**Background:**

The Functional Index of Hand Osteoarthritis (FIHOA) is a clinically and methodologically validated score used to assess functional impact in patients with hand osteoarthritis (OA). The aim of the study was to translate the FIHOA into classical Arabic, and to validate the psychometric properties of the translated version.

**Methods:**

The FIHOA was translated into Arabic (FIHOA-AR) according to cross-cultural adaptation guidelines. The FIHOA-AR was administrated to patients diagnosed with hand OA according to the criteria of the American College of Rheumatology (ACR). A 5-day test-retest reliability and internal consistency study was performed using the intra-class correlation coefficient (ICC) and the Cronbach’s alpha coefficient. External validity was measured by correlations between FIHOA-AR, hand pain visual analog scale (VAS) and the Health Assessment Questionnaire (HAQ).

**Results:**

The sample consisted of 101 patients with hand OA. The obtained ICC > 0.9 and Cronbach’s alpha of 0.93 indicated excellent reliability and internal consistency respectively. The evaluation of external validity showed strong correlation with hand pain VAS (*r* = 0.88, *p* < 0.001), and strong correlation with HAQ score (*r* = 0.86, *p* < 0.001).

**Conclusion:**

The FIHOA-AR is a reliable and valid score to assess functional disability in Arabic- speaking patients with hand OA.

## Background

Hand is a common location of osteoarthritis (OA). Clinical presentation varies from the absence of pain or dysfunction to a functional impact that can be severe. Hand OA is a main reason for frequent visits, not only because of the aesthetic discomfort but often also because of the functional impact which might be the main concern for many patients [1].

Literature presents a set of instruments designed for measurement of pain and functional capacity in patients with hand OA [2]. In this context, we can mention in particular, the Functional Index for Hand Osteoarthritis (FIHOA), which was validated by Dreiser, Maheu and colleagues in the early 1990s. This index was published for the first time in its English original version in 1995 [3]. It is a 10- item-questionnaire, scored on a semi-quantitative four-point scale (with responses ranging from 0 to 3). It is worth to emphasize that the precision of FIHOA has been well analyzed and widely documented. Thus, the responsiveness has been published in 2000 with the English version of the FIHOA [4]. One major conclusion that can be extracted from different analyses conducted on the FIHOA is the fact that the index can be considered as a reliable instrument. In addition, the FIHOA has the advantage to be sensitive to change, with a mean standardized response of 0.58 [4].

The FIHOA has been translated into 21 languages, which are accessible on the FIHOA website [5] as well as the original English version [see Additional file 1]. To be used internationally, a questionnaire must be translated into local languages and must also be culturally adapted. The aim of this study was to analyze and validate the translated Arabic version of the FIHOA.

## Methods

### Translation

The translation of FIHOA into Arabic followed the guidelines for cross-cultural adaptations of assessment tools [6]. These included: translation, back-translation and synthesis. Two independent bilingual translators whose mother tongue was Arabic prepared the Arabic translation from the French version of the FIHOA. One translator was a rheumatologist and was aware of the concept of our project and the second was a professional translator who was unaware about the study. The translators and one author (EM) assessed both translations and, after comparison, reached consensus. Then, a back-translation was elaborated by two other independent bilingual (French and Arabic) translators, who were uninformed about the original French version. The French version obtained by back-translation was compared with the original French version by a review committee to detect misinterpretations and nuances that might have been missed. The committee comprised the forwards and back translators, a methodologist, a linguistic and two rheumatologists. The final version, shown in Table 1 followed slight changes made by consensus and was then analyzed in this study.

**Table 1:**
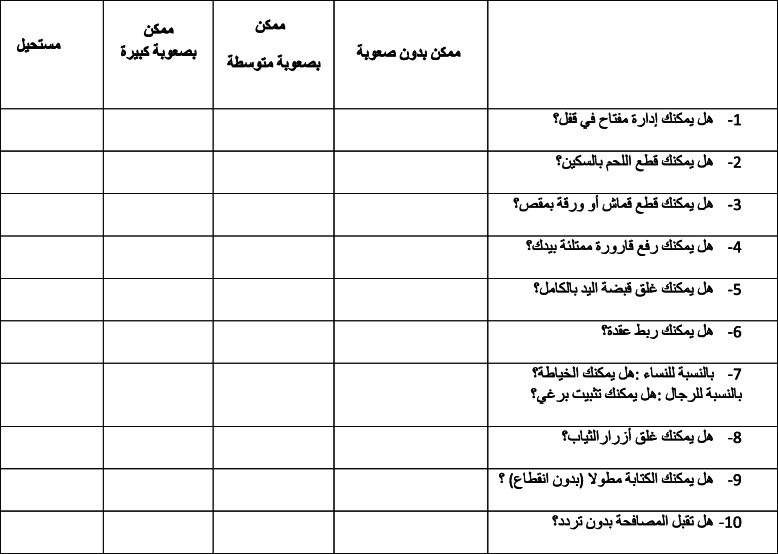
Arabic version of the Functional Index for Hand Osteoarthritis (FIHOA-AR)

### Patients

Our sample consisted of 101 Arabic-speaking patients who presented to the Department of Rheumatology of El Ayachi Hospital between March and September 2017. Participants had to meet the following inclusion criteria: (1) native Arabic speaking; (2) hand OA according to the American College of Rheumatology (ACR); and (3) ability to read, understand and answer the questionnaire. Exclusion criteria were: (1) patients with other rheumatic diseases; (2) illiterate patients.

During the first visit the Arabic version of FIHOA (FIHOA–AR) and others questionnaires were administered. All patients were asked to complete the FIHOA-AR again after 5 days. They all gave informed written consent. The study protocol was approved by the ethics committee for biomedical research of Mohammed V- Souissi University of Rabat.

### FIHOA-AR and other measures

#### FIHOA-AR

The FIHOA-AR is a scale that comprises 10 items with one sex specific question included. It can be administered during an interview or self-administered [3, 7]. The 10 items of the questionnaire are scored on a four-point scale for each described task or activity; the response categories are: “0 = possible without difficulty”, “1 = possible with slight/moderate difficulty”, “2 = possible with great difficulty” and “3 = impossible” [3].

#### Hand pain visual analogue scale

Patients were asked to assess the global pain in their hands which they had experienced during the last week, using a single Visual Analogue Scale (VAS) ranging from 0 (no pain) to 100 (greatest pain).

#### Health assessment questionnaire

Participants were requested to complete the Arabic version of the Health Assessment Questionnaire (HAQ) [8]. The HAQ explores eight domains of activity with two or three items in every domain. The response to every item is rated from 0 to 3, according to the difficulty encountered in the use of an instrument or the need for help by another person (“0 = without difficulty”, “1 = with some difficulty”, “2 = with much difficulty”, and “3 = unable to do”) [9]. The HAQ contains 10 items about hand function which were calculated separately.

### Statistical methods

Data were analyzed using SPSS_21.0. Test results are reported as significant if *p* < 0.05. Descriptive statistics and frequencies were computed. Data were expressed as frequencies and percentages for categorical variables. Mean and standard deviation (SD) or median and interquartile (IRQ) ranges were used for continuous and discrete data, respectively.

#### Test-retest reliability

Test-retest reliability refers to the stability of a score [10]. It was determined in our study, by interviewing the patients on two occasions separated by 5 days. The results were assessed by calculating the intra-class correlation coefficient (ICC) for the total score and for every item separately.

#### Internal consistency

This form of reliability refers to the extent to which items are interrelated [10]. It was determined through calculating Cronbach’s alpha coefficient. A high coefficient (0.70 or more) indicates that the items in a scale measure the same construct.

#### Internal construct and external validity

Factor analysis was used to assess internal construct validity. External construct validity was evaluated by calculating the correlation between FIHOA-AR, HAQ and pain VAS using Spearman’s correlation coefficient.

## Results

### Demographics and clinical characteristics

We recruited 101 patients (94 women and 7 men) with hand OA to complete the FIHOA-AR, HAQ and VAS. Their demographic and medical characteristics are listed in Table 2.

**Table 2:** Patients demographic and clinical characteristics

Variable	Total population *N* = 101	Symptomatic hand OA group (*N* = 32)	Non−/mildly-symptomatic hand OA group (*N* = 69)
Age (years)^1^	60.40 ± 8,1	61.63 ± 7.87	59.83 ± 8.19
Female gender^2^	94 (93,1%)	31 (96,9)	63 (91,3)
FIHOA-AR score (0–30)^3^	5 [1–11]	15 [9,25–18]	2 [0–5]
mHAQ (0–3)^1^	0.53 ± 0,35	0.89 ± 0.29	0.36 ± 0.22
Hand pain VAS (0–100)^3^	20 [1,5–40]	50 [40–60]	10 [0–20]
Right hand dominant^2^	101 (100%)	32 (100%)	69 (100%)

Values are given as mean ± standard deviation (^1^), frequency (^2^) or median and quartiles (^3^)

Female sex represented 93.1% of the analyzed cohort, with a right-handed dominance (100%). Regarding education, 63.5, 31.6 and 4.9% had completed primary school or non-formal education, secondary school and higher education respectively.

Patients were divided into non- or mildly symptomatic group (*N* = 69**)** and symptomatic group (*N* = 35**)** based on their VAS score. The cutoff was< 40 mm (non- or mildly symptomatic) and ≥ 40 mm (symptomatic group).

### Test–retest reliability

The FIHOA-AR was administered twice, 5 days apart, to all 101 patients. The median of the total score for the first assessment was 5 (IQR 1.0–11.0), and that of the second assessment was 4 (IQR 1.0–10.0) with no significant difference between the two measures (*p* = 0.16). In addition, Spearman’s rho between test and retest scores, was very near to 1 (0.99) and highly statistically significant (*p* < 0.001). We found the same result for single-item correlations, which were strong or very strong.

The average scores for items of the FIHOA-AR are reported in Table 3. Given the low percentage of men in our sample, the item 7 (gender-specific question) was analyzed without considering gender of patients. The item-item correlation values varied from 0.437 for items 1 and 5 to 0.709 for items 7 and 8 (Table 3). As already reported in previous studies [3, 7], the index may induce some redundancy. We didn’t note any refusal or missing items.

**Table 3:** Test-retest reliability of the FIHOA-AR

FIHOA-AR test – FIHOA-AR retest	Test	Retest	Spearman’s rho	ICC	95% CI
FIHOA-AR test – FIHOA-AR retest	67,327 ± 6,76	66,238 ± 6,81	0,990	0,994	0,99-0,996
Item 1 – Item 1 retest	0.60 ± 0.813	0.59 ± 0.815	0.975	0.962	0945–0,974
Item 2 - Item 2 retest	1 ± 0.860	0,98 ± 0.848	0.987	0.987	0,980-0,991
Item 3 - Item 3 retest	0.72 ± 0.862	0.66 ± 0.803	0.972	0.945	0,919-0,963
Item 4 - Item 4 retest	0.44 ± 0.684	0.39 ± 0.632	0,907	0.899	0,854-0,931
Item 5 - Item 5 retest	0.96 ± 1.157	0.95 ± 1.161	0,991	0.996	0,995-0,998
Item 6 - Item 6 retest	0.49 ± 0.730	0.48 ± 0.769	0,863	0.902	0,858-0,933
Item 7 - Item 7 retest	0.77 ± 0.947	0.74 ± 0.945	0,963	0.973	0,960-0,981
Item 8 - Item 8 retest	0.57 ± 0.792	0.6 ± 0.801	0,899	0.930	0,898-0,952
Item 9 - Item 9 retest	0.5 ± 0.743	0.56 ± 0.780	0,779	0.806	0,725-0,865
Item 10 - Item 10 retest	0.68 ± 0.848	0.66 ± 0.816	0,960	0.978	0,937-0,971

### Internal consistency

We calculated Cronbach’s alpha for the overall FIHOA-AR. A value of 0.93 suggested very good internal coherence among the items of the questionnaire. Adjusted item–total correlation for all items was efficient (0.692–0.840) and statistically significant (*p* < 0.01). As shown in Table 4, Cronbach’s alpha after deleting 1 item ranged from 0.923 to 0.934 which indicated strong internal consistency.

**Table 4:** Internal consistency of the FIHOA-AR

Items	Mean	Scale mean if item is deleted	Scale variance if item is deleted	Adjusted item-total Spearman’s rho	Item-item correlation Spearman’s rho	Cronbach’s alpha if item is deleted
Item 1	0.60 ± 0.813	61,287	37,653	0,735	0.437–0.640	0,927
Item 2	1 ± 0.860	57,327	36,978	0,840	0.489–0.692	0,926
Item 3	0.72 ± 0.862	60,099	37,130	0,818	0.541–0.672	0,927
Item 4	0.44 ± 0.684	62,970	39,431	0,692	0.438–0.668	0,931
Item 5	0.96 ± 1.157	57,723	34,938	0,761	0.437–0.679	0,934
Item 6	0.49 ± 0.730	62,475	38,028	0,735	0.489–0.679	0,925
Item 7	0.77 ± 0.947	59,604	35,598	0,801	0.460–0.709	0,923
Item 8	0.57 ± 0.792	61,584	37,235	0,761	0.504–0.709	0,924
Item 9	0.5 ± 0.743	62,376	38,283	0,725	0.438–0.632	0,927
Item 10	0.68 ± 0.848	60,495	37,748	0,740	0.460–0.664	0,929

Values are given as mean ± standard deviation or range

Spearman’s rho indicates Spearman’s correlation coefficient

### Internal construct validity

**Fig. 1:**
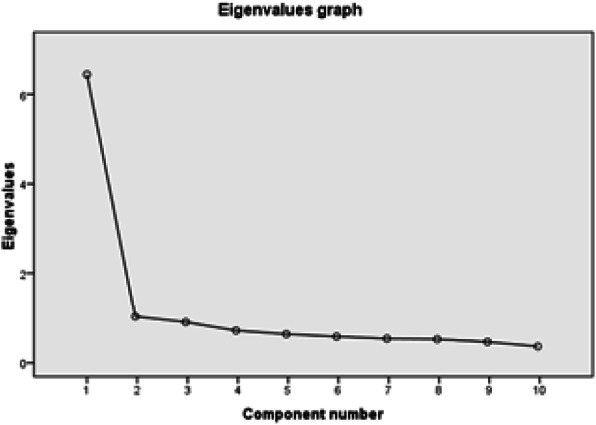
Scree plat of FIHOA-AR

In order to assess the internal structural validity of FIHOA-AR, we used factor analysis. The Kaiser–Olkin value was 0.901, which confirmed that the sample size was sufficient for the use of factor analysis. Value of χ2 (produced by Bartlett’s test of sphericity) equal to 741.83 (*p* < 0.01), suggested that the factor model was appropriate. Based on the total variance, we confirmed that the selection of four components explains 83.65% of the overall variance. Taking factors separately, we mentioned that the first factor accounted for 64.42%, the second accounted for 7.87%, the third accounted for 6.625% and the fourth factor accounted for 4.737% of the total variance. In addition, after four component extraction, the scree plot became nearly flat. However, by applying the “eigenvalue> 1” rule, only 2 factors could be considered (Fig. 1). The first factor, which explained 64.42% of the total variance captured patient’s general incapacity in executing activities. The first component was strongly correlated with the total score. Decision about rotation transformation can be considered using Varimax rotation, which showed that the first factor is composed by the following four items: Item 6 (Can you tie a knot?), item 9 (Can you write for a long period of time?), item 8 (Can you fasten buttons?) and item 7 (Can you sew? or Can you use a screwdriver?). The first factor thus included activities that require the ability to use one’s fingers precisely.

The second factor, explaining 7.87% of the total variance, was composed of item 4 (Can you lift a full bottle with the affected hand?) and item 2 (Can you cut meat with a knife). The second factor therefore captured items that involved a certain amount of grip strength.

### External construct validity

To evaluate external construct validity, we calculated Spearman’s rho correlation coefficient between the total FIHOA-AR, VAS hand pain and HAQ. Strong and significant correlation (*r* = 0.88, *p* < 0.001) was observed between FIHOA-AR and VAS. For HAQ and FIHOA-AR, the correlation was again strong and significant (0.86, *p* < 0.001).

## Discussion

Results from this study indicate that the FIHOA-AR is a valid and reliable instrument to measure functional disability in Arabic-speaking patients with hand OA. Cultural adaptation was not necessary because of the universal character of each item, as these can easily be understood in any language and culture. The brevity and simplicity of using FIHOA contributes to make the instrument acceptable to both patients and doctors in clinical practice. We used a validation methodology similar to that followed for the validation of the others translated versions of the same questionnaire [4, 7].

The psychometric properties of the Arabic questionnaire were highly acceptable, with internal consistency of 0.93 for the total scale. This result indicates strong internal coherence between the test items. The test-re-test reliability for all items was *r* = 0.99, which indicates that patients’ perception of the items remained stable over time. The overall internal consistency (0.93) is similar to that reported in previous publications; 0.89 was reported in the Persian version [11], 0.87 for the Italian version [12] and 0.88 for the Korean version [13].

Factor analysis of FIHOA-AR indicated that it is a two-dimensional questionnaire. Dreiser and colleagues had already emphasized the non-unidimensionality of the original version of FIHOA [3]. We extracted two components for the FIHOA-AR: a certain degree of grip strength and accurate finger movement with wrist stability. This was observed also in the Persian translation [11]. The FIHOA was reported as a one-dimensional index in the Norwegian version of the FIHOA [14]. Gandini et al. captured three components in the Italian FIHOA: accurate and proper finger movement, the ability of the wrist to support a certain amount of strength, and certain grip strength level while tightening the hand [12]. Ahn GY et al. extracted four components in the Korean FIHOA: “The 1^st^ 3 components describe hand function and the 4^th^ factor embodies the social nuance, as well as the functioning of hand OA itself” [13].

External construct validity was good; we found strong correlation between FIHOA-AR and HAQ (*r* = 0.86, *p* < 0.001) and between FIHOA-AR and hand pain VAS (*r* = 0.88, *p* < 0.001).

Our study has some limitations. The first is that our study population comprised only Moroccan patients. However the simplicity of items and the socio-cultural similarity between most Arab countries suggests its acceptability by Arab patients in other countries. The use of classical written Arabic makes the FIHOA-AR readable and understandable by all educated Arab individuals who can read classical Arabic, regardless of their country. Second, we tested the FIHOA-AR only on educated patients, because illiterate people would be unable to understand classical Arabic and to respond to item 9 (Can you write for a long period of time?). For most of the elderly population, translation to local languages would be more appropriate. Third, our patient population was mainly female and was entirely right-handed. Hence, the results might not be generalized to the entire population of patients with hand OA. Another limitation was that the test-retest reliability was undertaken 5 days apart. This short interval was choose to minimize changes in the patient’s overall health status; however, it can help patients to remember their previous responses than if this was undertaken over a longer period such as 2–4 weeks. Finally, we did not evaluate the external validity of the FIHOA-AR with the *AUStralian CANadian Osteoarthritis Hand Index* (AUSCAN) which is another instrument used to evaluate pain and disability in patients with hand OA [15]. Unfortunately, there is no validated Arabic translation of the AUSCAN.

## Conclusion

The Arabic version of FIHOA showed good psychometric properties when used with a sample of Moroccan patients with hand OA. Its use in clinical practice would be interesting to assess the functional disability and outcomes and for improving management of hand OA patients. In addition, the scale may easily be used as a main outcome for assessing functional impairment in clinical trials of therapies for hand OA.

## Supplementary information

**Additional file 1.** The original English version of FIHOA.

## Data Availability

The datasets used and/or analysed during the current study are available from the corresponding author on reasonable request.

## Abbreviations

ACR: American college of rheumatology
AUSCAN: AUStralian CANadian osteoarthritis hand index
FIHOA: Functional index for hand osteoarthritis
FIHOA-AR: Arabic version of FIHOA
HAQ: Health assessment questionnaire
ICC: Intra-class correlation coefficient
IQR: Interquartile range
OA: Osteoarthritis
SD: Standard deviation
VAS: Visual analogue scale

## References

[1] Kloppenburg M, Kwok W (2012). Hand osteoarthritis—a heterogeneous disorder. Nat Rev Rheumatol.

[2] Poole JL (2011). Measures of hand function: Arthritis Hand Function Test (AHFT), Australian Canadian Osteoarthritis Hand Index (AUSCAN), Cochin Hand Function Scale, Functional Index for Hand Osteoarthritis (FIHOA), Grip Ability Test (GAT), Jebsen Hand Function Test (JHFT), and Michigan Hand Outcomes Questionnaire (MHQ). Arthritis Care Res (Hoboken).

[3] Dreiser RL, Maheu E, Guillou GB, Caspard H, Grouin JM (1995). Validation of an algofunctional index for osteoarthritis of the hand. RevRhum (Engl Ed).

[4] Dreiser RL, Maheu E, Guillou GB (2000). Sensitivity to change of the functional index for hand osteoarthritis. Osteoarthritis Cartilage.

[5] FIHOA [Internet]. https://fihoa.net/score. Accessed 6 July 2018.

[6] Beaton DE, Bombardier C, Guillemin F, Ferraz MB (2000). Guidelines for the process of cross-cultural adaptation of self-report measures. Spine (Phila Pa 1976).

[7] Wittoek R, Cruyssen BV, Maheu E, Verbruggen G (2009). Cross-cultural adaptation of the Dutch version of the functional index for hand osteoarthritis (FIHOA) and a study on its construct validity. Osteoarthr Cartil.

[8] Bourazzak FE, Benbouazza K, Amine B, Bahiri R (2008). Psychometric evaluation of a Moroccan version of health assessment questionnaire for use in Moroccan patients with rheumatoid arthritis. Rheumatol Int.

[9] Fries JF, Spitz PW, Young DY (1982). The dimensions of health outcomes: the health assessment questionnaire, disability and pain scales. J Rheumatol.

[10] Cronbach LJ (1947). Test “reliability”: its meaning and determination. Psychometrika.

[11] Kordi Yoosefinejad A, Motealleh A, Babakhani M (2017). Evaluation of validity and reliability of the Persian version of the functional index of hand osteoarthritis. Rheumatol Int.

[12] Gandini F, Giannitti C, Fattore G, Giordano N, Galeazzi M, Fioravanti A (2012). Validation of an Italian version of the functional index for hand osteoarthritis (FIHOA). Mod Rheumatol.

[13] Ahn GY, Cho S-K, Cha SJ (2018). Cross-cultural adaptation and validation of the Korean version of the functional index for hand osteoarthritis (FIHOA). Int J Rheum Dis.

[14] Moe RH, Garratt A, Slatkowsky-Christensen B (2010). Concurrent evaluation of data quality, reliability and validity of the Australian/Canadian osteoarthritis hand index and the functional index for hand osteoarthritis. Rheumatology (Oxford).

[15] Bellamy N, Campbell J, Haraoui B (2002). Dimensionality and clinical importance of pain and disability in hand osteoarthritis: development of the Australian/Canadian (AUSCAN) osteoarthritis hand index. Osteoarthr Cartil.

