# Supplementary material for: Cross-cultural adaptation and validation of the Arabic version of the functional index for hand osteoarthritis
Source: BMC Musculoskelet Disord. 2020 Jun 19;21:390. doi: 10.1186/s12891-020-03418-8 (PMC7305589; doi:10.1186/s12891-020-03418-8)
Supplement: Supplementary file 1 — Additional file 1. The original English version of FIHOA. [file 12891_2020_3418_MOESM1_ESM.pdf]

The original English version of FIHOA

	Possible without difficulty	Possible with slight difficulty	Possible with important difficulty	Impossible
1-Are you able to turn a key in a lock?				
2-Are you able to cut meat with a knife?				
3-Are you able to cut cloth or paper with a pair of scissors?				
4-Are you able to lift a full bottle with the hand?				
5-Are you able to clench your fist?				
6-Are you able to tie a knot?				
7-*For women* - Are you able to sew?<br>*For men* - Are you able to use a screwdriver				
8-Are you able to fasten buttons				
9-Are you able to write for a long period of time (10 mn)?				
10-Would you accept a handshake without reluctance?				

The Functional Index for Hand Osteoarthritis (FIHOA), which was validated and published by Dreiser, Maheu and colleagues for the first time in its English original version in 1995 [3]. The FIHOA has been translated into 21 languages, which are accessible on the FIHOA website [5] as well as the original English version.
